# Positional functionalizations of metal–organic frameworks through invasive ligand exchange and additory MOF‐on‐MOF strategies: A review

**DOI:** 10.1002/smo.20240002

**Published:** 2024-05-20

**Authors:** Daeyeon Lee, Sangho Lee, Isaac Choi, Min Kim

**Affiliations:** ^1^ Department of Chemistry Chungbuk National University Cheongju Republic of Korea

**Keywords:** metal–organic frameworks (MOFs), MOF‐on‐MOF multifunctional materials, porous coordination polymers (PCPs), post‐synthetic exchanges (PSEs)

## Abstract

Metal–organic frameworks (MOFs) represent a unique class of porous materials with tremendous potential for diverse applications. A key factor contributing to their versatility is their ability to precisely introduce functional groups at specific positions within pores and crystals. This review explores two prominent strategies for achieving the positional functionalization of MOFs: post‐synthetic ligand exchange (PSE) and MOF‐on‐MOF. In PSE, the existing ligands within solid‐state MOFs can be selectively replaced by the desired functional groups in solution through ligand dynamics. This invasive functionalization provides a flexible approach to fine‐tuning the surface of the MOFs with the target functionality. Conversely, MOF‐on‐MOF strategies are additive methodologies involving the controlled growth of one MOF layer onto another. The functionality of the core and shell (or surface) can be independently controlled. This review critically examines the examples, strengths, limitations, and applications of these strategies, emphasizing their significance in advancing the field of MOF functionalization and paving the way for tailored multifunctional materials with precise and specific properties.

## Graphical abstract

Positional Functionalization in Metal–Organic Frameworks (MOFs): Delve into the precision of functionalization strategies within MOFs by exploring post‐synthetic ligand exchange (PSE) and MOF‐on‐MOF techniques. PSE selectively replaces existing ligands, allowing flexible surface tuning, while MOF‐on‐MOF methods involve controlled layer growth for independent core and surface functionalities. This review critically assesses examples, strengths, limitations, and applications, highlighting their pivotal role in advancing MOF functionalization for tailored multifunctional materials.

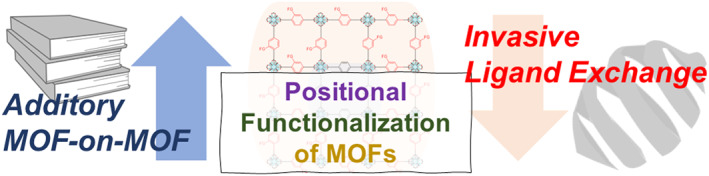

## 1 INTRODUCTION

Metal–organic frameworks (MOFs) are three‐dimensional, coordination‐based, highly porous, organic–inorganic hybrid materials. They are fabricated by the formation of repeating coordination bonds between metal clusters (secondary building units [SBUs]) and multitopic organic ligands.[^1^, ^2^, ^3^] The structural aspects of MOFs exhibit significant diversity owing to the wide range of metal sources and ligands, along with their coordination modes. Furthermore, the ligands in MOFs can be functionalized with various groups, such as amino groups, hydroxyl groups, and halogens, adding to their versatility and functions. This functionalization enables identical frameworks compared to the parent MOFs that lack functionalized ligands, considerably expanding the diversity of MOFs.[^4^, ^5^, ^6^] The pores of MOFs can be decorated with functional groups on ligands and/or functional moiety in the pore.^[7]^

Organic functional groups play a crucial role in the tailoring of MOFs for specific applications. For instance, MOFs incorporating amino groups exhibit enhanced affinity for carbon dioxide compared to their non‐functionalized counterparts.^[8]^ Similarly, MOFs functionalized with alkyl bromides can be utilized for the direct polymerization with monomers via radical polymerization.^[9]^ The strategic incorporation of diverse organic functional groups into MOF pores enables tailored applications, and the precise positioning of these functional groups is critical for achieving the desired MOF properties and their applications.[^10^, ^11^]

Two main strategies have been developed to introduce the desired functional groups into the MOF pores by controlling the timing of functionalization. The first strategy is pre‐functionalization, which involves direct synthesis with a functionalized ligand. For instance, amino‐functionalized MOFs can be obtained by combining metal salts with amino‐functionalized ligands. The second approach is post‐synthetic modification (PSM), where the desired functional group is installed on the MOFs after their formation through a solid‐state transformation.[^12^, ^13^, ^14^] For example, amide functionalities can be introduced into MOFs through the solid‐state acylation of amino‐functionalized MOFs. PSM is a powerful strategy for incorporating metal‐chelating groups (which may not be suitable for MOF formation), thermally unstable groups (unsuitable for solvothermal conditions), and a series of functional groups (e.g., amide functionalities with various substituents) into MOFs.^[15]^

MOFs also serve as excellent platforms for the incorporation of multiple functionalities into single pores. The mixed‐ligand strategy enables the formation of homogeneous mixed solids by combining ligands with the same coordination sites, but different functional groups. For instance, by simply blending amino‐functionalized terephthalic acid (i.e., 2‐amino‐benzene‐1,4‐dicarboxylic acid, BDC‐NH_2_) and bromo‐functionalized terephthalic acid (i.e., 2‐bromo‐benzene‐1,4‐dicarboxylic acid, BDC‐Br) during the solvothermal synthesis, isoreticular MOFs can be obtained, incorporating homogeneously mixed functional groups through the entire MOF particle.[^16^, ^17^, ^18^, ^19^, ^20^]

The mixed‐ligand strategy, based on MOF formation from a homogeneous ligand solution, often leads to the incorporation of functional groups in random positions, resulting in mainly random‐mixture solids. In contrast, the PSM strategy relies on solid‐state transformation, providing an opportunity for position‐specific functionalization within crystalline materials. Notably, post‐synthesis exchange (PSE) is a promising approach for inserting target functional groups at specific positions on MOF particles, primarily on external surfaces.[^21^, ^22^]

**FIGURE 1:**
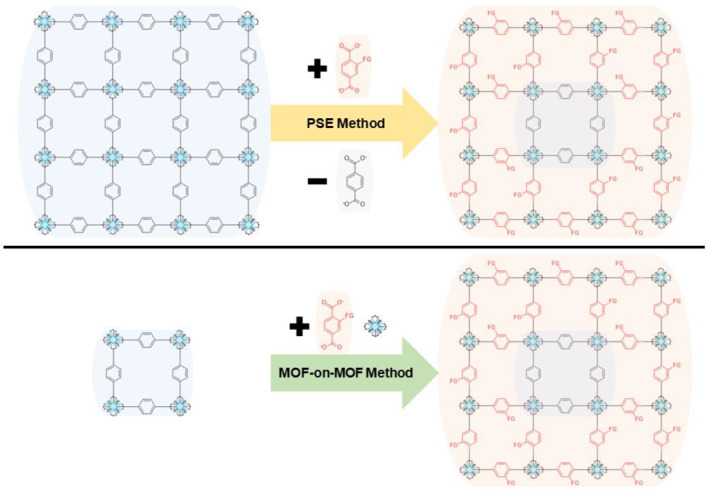
Two main strategies for positional functionalization of MOFs: invasive PSE method (top) and the additory MOF‐on‐MOF method (bottom).

In PSE, the two main MOF components (ligands and metal ions) can be exchanged with external ligands or metal ions present in the solution (Figure 1). For example, when a bromo‐functionalized MOF is incubated in a solution containing amino‐functionalized ligands, an exchange occurs between the amino‐functionalized ligand in the solution and the bromo‐functionalized ligand in the solid MOF. Consequently, the desired functional group installation can be achieved through PSE using the target ligand solution. Moreover, because of the similar sizes of the ligands involved, the diffusion of the target ligand is considerably restricted. This restricted diffusion leads to an exchange process that predominantly occurs from the surface to the core of the MOF particles, enabling directional control over the installation of functional groups.[^23^, ^24^, ^25^, ^26^, ^27^]

Recently, the MOF‐on‐MOF strategy has garnered considerable attention as a means of developing multifunctional MOFs. This strategy involves the growth of secondary MOFs on the surface of core MOFs, resulting in core–shell type MOFs, where the functionality in the core and the shell can be isolated (Figure 1). Although the MOF‐on‐MOF strategy cannot be applied to all combinations of known MOFs, substantial progress has been made in the synthesis of various core–shell MOFs with similar and different topologies.[^28^, ^29^, ^30^, ^31^, ^32^, ^33^]

In this review article, we focus on recent advances in the positional functionalization of MOFs. Our main emphasis is on exploring the differences in the functional groups within single particles (or single crystals) with the same coordination modes. Both the PSE and MOF‐on‐MOF strategies are discussed in terms of their potential for functional group combinations and mechanistic aspects. Furthermore, we classify the applications of micro‐tuned MOFs to highlight the advantages of the positional control of functional groups in MOFs. We firmly believe that the directional and positional control of the functional groups in MOFs, along with their practical preparation, represents a crucial advantage of crystalline, solid‐state, and functional MOFs over other solid materials. Overall, this review aims to provide insights into the recent progress in manipulating functional groups within MOFs, shedding light on promising directions and potential applications in this fascinating and rapidly evolving field.

## 2 FUNCTIONALIZATION OF MOFs THROUGH PSE APPROACH

### 2.1 Functionalization of MOFs through standard PSE

**FIGURE 2:**
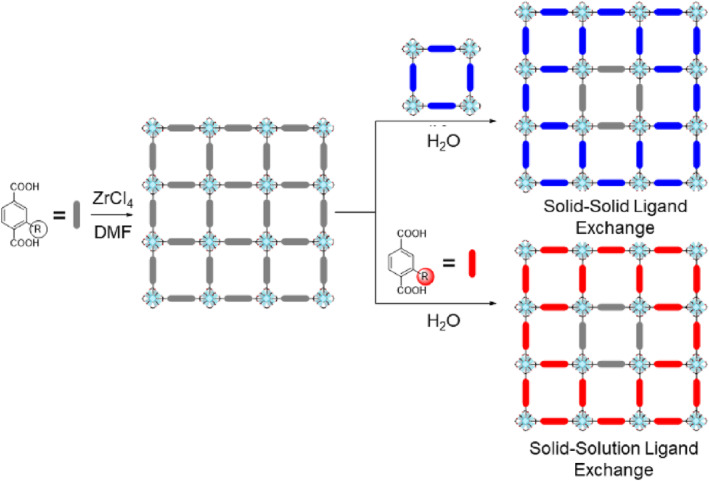
PSE process for the functionalization of MOFs.

The MOF‐coordination nature enables the exchange of ligands in the solid state with the external ligands in solution. The desired functionalization can be achieved through this post‐exchange process not only for ligands, but also for metal cations.[^24^, ^25^, ^26^, ^27^, ^34^, ^35^, ^36^, ^37^] Choe and colleagues were among the pioneers in demonstrating the application of PSE in pillared MOFs. They introduced nitrogen‐coordinating ligands, such as 4,4′‐bipyridyl, into pre‐synthesized MOFs at the pillar position through solid‐solution exchange, and a step‐wise MOF synthesis method was successfully facilitated through ligand exchange with the nitrogen‐coordinating ligand.[^38^, ^39^, ^40^] In 2012, the Cohen group reported an extended PSE process with dicarboxylic acid ligands on Zr‐based MOFs for solid–solid exchange (through solution), and solid–solution exchange (Figure 2).^[21]^ Initial investigations focused on ligand exchange between two distinct MOFs, UiO‐66‐NH_2_ and UiO‐66‐Br, using a particle analyzer and aerosol time‐of‐flight mass spectrometry (ATOFMS). Next, this phenomenon was extended to the solid–liquid phase ligand exchange between non‐functionalized MOFs and the target ligand solution. Hydroxy, dihydroxy, and azide groups were successfully incorporated into a nonfunctionalized MOF (UiO‐66). Importantly, the PSE method overcomes the limitations associated with the PSM approach, enabling the incorporation of diverse functional groups beyond those initially present in the parent MOF. Consequently, the development of PSE has expanded the possibilities for MOF synthesis with multiple functional groups integrated within a single framework. In the following work by Cohen's group, the PSE process was applied to a diverse range of robust MOFs, including Zr‐, Ti‐, and Hf‐based UiO series, Al‐, Fe‐, and In‐based MIL series (MIL = Materials of Institute of Lavoisier), and zeolitic imidazole frameworks (ZIFs).^[22]^ In addition to the diversity of frameworks, the exchange of metal cations between solid and solid and solid solutions was also confirmed using ATOFMS analysis.

Multiple research teams have conducted detailed and in‐depth analyses of the PSE process. In 2019, Ameloot and co‐workers conducted a study aimed at investigating the molecular‐level processes of PSE of MOFs.^[41]^ Through time‐resolved monitoring of the PSE in Zr‐based UiO‐66, the PSE kinetics, changes in cluster coordination, the ratio of outgoing and incoming linkers, and the influence of framework defects and solvent interactions were investigated. Consequently, the crucial role of the solvent (e.g., methanol) in generating and stabilizing linker defects within the MOF was demonstrated. However, the presence of defects at the initiation of the PSE did not significantly affect the overall exchange process. Finally, the uniform distributions of the original ligand (from the mother MOFs) and the new ligand from PSE were confirmed through solid and solution analyses. This comprehensive investigation of the kinetics and mechanisms of PSE in UiO‐66 sheds light on the dynamic behavior of ligand exchange at the molecular level.

Computational approaches for understanding the PSE process in MOFs were developed by Tsai et al. using density functional theory (DFT) calculations.^[42]^ The acid‐catalyzed associative substitution mechanism for PSE was revealed through DFT calculations, and a step‐by‐step pathway for functionalized ligand installation was suggested. Furthermore, this computational approach indicated that the PSE rate is influenced by the relative acid–base properties of carboxylic acids (i.e., ligands of MOFs and modulators).

**FIGURE 3:**
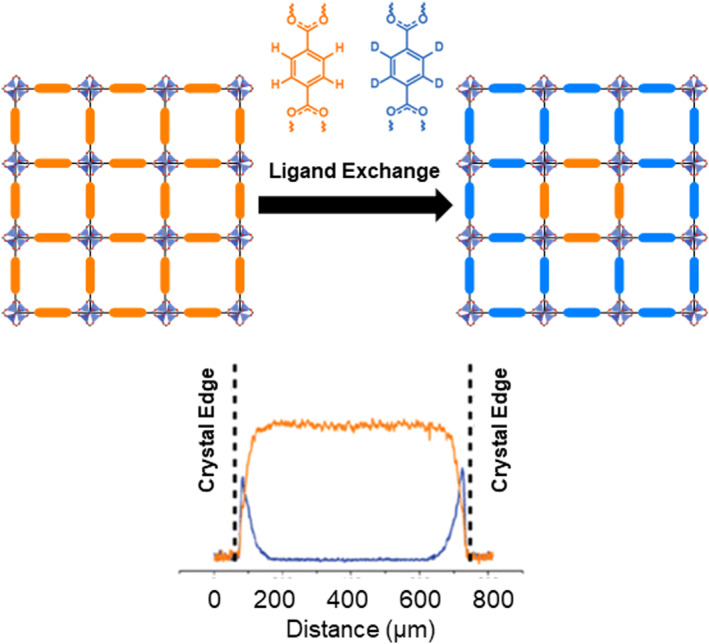
Visualization of PSE on MOF surfaces through C–D bonds. Adapted with permission (parts of figures) from Reference 43. Copyright 2017. American Chemical Society.

Visualized experimental approaches for understanding the PSE behavior have also been attempted. In 2017, Matzger et al. conducted an in‐depth investigation into the mechanism and specific localization of ligand exchange in MOF PSE (Figure 3).^[43]^ The research team focused on examining the distribution of linkers and recognizing their potential impact on the interactions with guest molecules. Zn‐based MOF‐5 with BDC ligands was used as the parent MOF, and deuterated BDC‐*d*_4_ was employed as the exchange ligand. For the main experiment, PSE was performed on single crystals of MOF‐5, followed by sectioning using an ultramicrotome. The resulting sliced surfaces were examined using a Raman microscope to check the position of BDC‐*d*_4_ in the crystal. Analysis of the Raman maps revealed that PSE primarily occurred at the surface, leading to the formation of a core–shell structure that extended inward toward the center (Figure 3). Notably, no exchanged ligands (BDC‐*d*_4_) were observed within the core, even with prolonged reaction time or increased temperature. This observation further confirmed that surface‐mediated ligand exchange was the dominant process in the PSE. Additionally, this positional functionalization was observed not only in Zn‐based MOF‐5, but also in Zn‐based UMCM‐8 and Zr‐based UiO‐66, indicating its occurrence across different frameworks.

In a subsequent study by the Matzger group, the solvent effects of PSE on MOFs were investigated using Raman mapping of the C–D bond.^[44]^ Thirty solvents, including amides, aldehydes/ketones, ethers, esters, carbonate esters, DMSO, sulfolane, and acetonitrile, were comprehensively investigated to determine the shell thickness and degree of exchange. Specifically, four scenarios were considered: high exchange rate with a thick shell, high exchange rate with a thin shell, low exchange rate with a thick shell, and low exchange rate with a thin shell. The observations demonstrated the occurrence of selective ligand exchange on the surface, providing valuable insights into the solvent‐dependent effects of PSE on MOFs.

Further investigations to reveal the distribution of the exchanged ligands in Zr‐based UiO‐66 MOFs were performed by the Benz group.^[45]^ Both UiO‐66‐I and UiO‐66‐I_2_ were synthesized using the PSE strategy and analyzed by *X*‐ray photoelectron spectroscopy. The authors verified that the selective incorporation of the desired ligands throughout the MOF particles or on the surface of the MOF particles could be achieved by controlling the reaction time. Shorter reaction time led to selective surface installation of the target ligand. Therefore, the functionality in the core and on the surface could be independently controlled by the PSE strategy.

**FIGURE 4:**
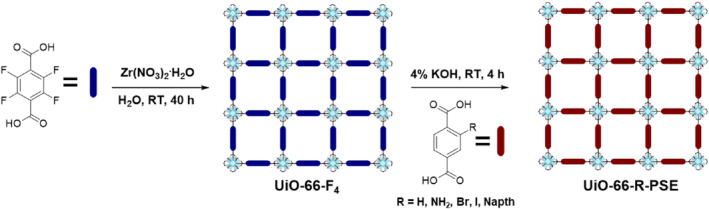
Full functionalization of MOFs using PSE and electron‐efficient ligands.

Full target ligand installation through PSE was achieved by Cohen et al. using environmentally friendly and water‐stable approaches (Figure 4).^[46]^ The tetrafluoro‐functionalized, Zr‐based MOF, UiO‐66‐F_4_ was selected for the hydrothermal synthesis of the parent MOFs. The choice of the parent MOFs was based on the hypothesis that the presence of four electron‐withdrawing groups on the BDC ligand weakens its coordination with the Zr nodes. This weakening of the coordination facilitates easier ligand detachment during PSE, leading to enhanced ligand exchange efficiency. Notably, this study achieved a ligand‐exchange rate of approximately 95% within 4 h, resulting in a significant reduction in the time required for PSE.

### 2.2 Functionalization of defective MOFs through PSE

Early studies by Behrens et al. revealed the role of organic acid additives in Zr‐based MOFs.^[47]^ The competition between coordination bond formation and Zr clusters between the BDC molecules and organic acid additives controlled the crystallization kinetics to form highly crystalline materials. The use of acid modulators (such as acetic acid and benzoic acid) plays a crucial role in facilitating the generation of defects in the MOF without compromising its crystallinity. These defects can be classified into two primary types: organic linker and SBU defects.[^48^, ^49^, ^50^]

The introduction of structural defects into MOFs is directly related to the enhancement of their adsorption properties and catalytic activities. At the same time, PSE functionalization was also correlated with the existence of structural defects. In 2016, Lillerud et al. investigated enhanced ligand exchange in defective MOFs.^[51]^ It was revealed that preferential ligand installation occurred at the defect site rather than the PSE process between the parent and target ligands. The number of defects was successfully controlled by adjusting the strength and stoichiometry of the acid.

**FIGURE 5:**
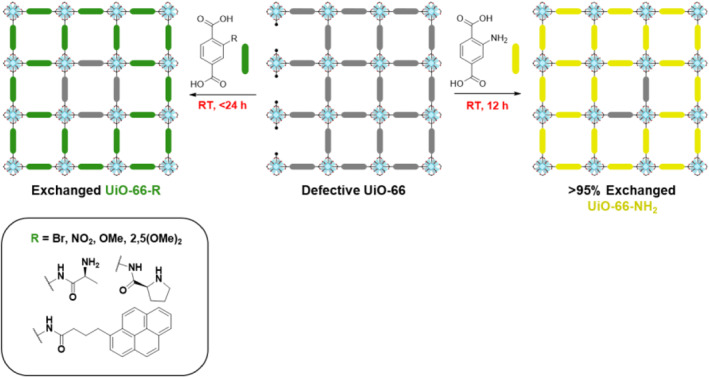
Functional group loading on the defect sites of MOFs and PSE.

Kim et al. further investigated the correlation between defects in MOFs and the PSE strategy (Figure 5).^[23]^ Three defect‐controlled Zr‐based UiO‐66 MOFs were prepared, and the most defective MOFs (from a trifluoroacetic acid modulator) achieved a remarkable PSE rate of target ligand of approximately 95% within 12 h. In addition, triple‐layered core–shell MOFs were synthesized through consecutive PSE steps. This approach facilitated the selective incorporation of desired functional groups in a controlled manner. Interestingly, during the second PSE step, it was experimentally observed that only the ligands introduced in the first PSE step underwent exchange, confirming the directional nature of PSE from the surface toward the interior. Moreover, this study demonstrated the improved efficiency of PSE, leading to the development of methodologies for the efficient and rapid incorporation of challenging functional groups, such as pyrene groups or amino acid moieties, which were previously difficult to introduce. Overall, the introduction of defect sites into the MOFs accelerated the overall rate and quantities of PSE. However, at the same time, the positional controls in target ligand installations were restricted as the structural defects were homogeneously distributed throughout the MOF particles.

Independently, Andreoli et al. introduced N‐rich ligands (e.g., amino and pyridine groups) into defective Zr‐based UiO MOFs to enhance their carbon dioxide capturing abilities.^[52]^ From the various defective MOFs, UiO‐66 from solvothermal synthesis with a formic acid modulator displayed the highest porosity and pore volume. For the PSE target, both BDC‐NH_2_ and ortho‐amino benzoic acid exhibited the best performance for carbon dioxide capturing ability. In this study, the target ligand was mainly installed on the defect sites of formic acid; therefore, true ligand exchange (between the parent ligand and the target ligand) was not claimed, and this process was called post‐synthesis defect exchange. Moreover, the positional installation of the target ligand was not sufficiently investigated.

### 2.3 Positional functionalization of MOFs through PSE

**FIGURE 6:**
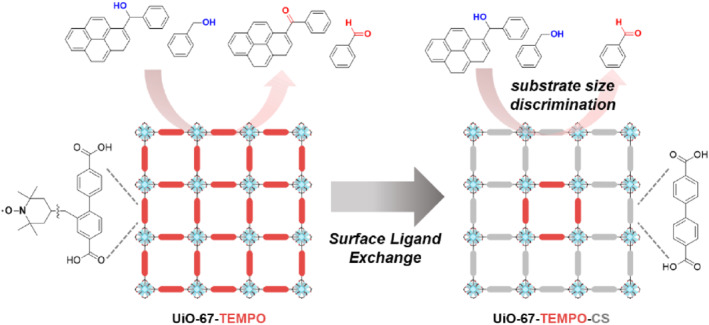
Surface deactivation of MOF‐based catalysts via PSE.

The directional installation of target ligands at the surface of MOFs enables the easy preparation of core–shell‐type, multi‐functional, porous materials through the PSE strategy. In 2020, Kim et al. reported a pioneering study of PSE on MOFs to enhance the substrate size selectivity for aerobic oxidation (Figure 6).^[53]^ Initially, a Zr‐based UiO‐67‐TEMPO MOF was synthesized, and the active TEMPO radical was utilized for the aerobic oxidation of alcohols to their corresponding aldehydes or ketones.[^54^, ^55^] This parent MOF (UiO‐67‐TEMPO) showed low substrate‐size selectivity because aerobic oxidation could occur at the TEMPO radicals on the surface of the MOFs.

The PSE strategy was then utilized to selectively introduce nonfunctionalized biphenyl dicarboxylic acid (BPDC) ligands without TEMPO radical species exclusively on the surface, resulting in the synthesis of UiO‐67‐TEMPO‐CS. Since the reactivity of the surface was removed by the PSE, only the catalytic reaction sites remained in the core. Therefore, this PSE type “surface‐deactivation” strategy effectively prevented larger alcohols from undergoing catalytic reactions within the MOF catalysts. The successful demonstration of surface deactivation using PSE opens new possibilities for designing MOFs with tailored catalytic properties. Although previous PSE strategies for MOFs were mainly based on the additional functionalization of MOFs, the present study highlights the passivation of functionality on MOFs through PSE. In addition, this study provides valuable insights into the potential of PSE as a tool for the precise engineering of the surface properties of MOFs.

**FIGURE 7:**
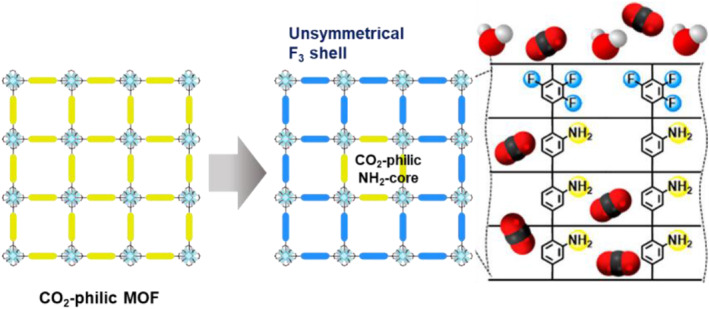
Formation of a hydrophobic layer on MOFs via PSE. Adapted with permission (parts of figures) from Reference 56. Copyright 2020. American Chemical Society.

Positional functionalization through PSE was then applied for the selective separation of molecules. Kim et al. applied the core–shell MOFs obtained from PSE for carbon dioxide capture under humid conditions (Figure 7).^[56]^ In general, amino‐functionalized UiO‐66‐NH_2_ is a good candidate for carbon dioxide capture, but its performance decreased under humid conditions. Therefore, hydrophobic shields are required to prevent water sorption and maintain the carbon dioxide capturing ability. Hence, core–shell type UiO‐66‐(NH_2_) (F_3_)‐CS was prepared through the PSE process of UiO‐66‐NH_2_ with the trifluoro ligand BDC‐F_3_. In addition, the homogenously mixed MOFs UiO‐66‐(NH_3_) (F_3_) were synthesized using the solvothermal conditions of the ligand mixtures (BDC‐NH_2_ and BDC‐F_3_) as a control sample. Then, the CO_2_/N_2_ separation performance was evaluated under both humid and dry conditions. The results demonstrated that the positionally functionalized UiO‐66‐(NH_2_) (F_3_)‐CS exhibited a significantly higher CO_2_/N_2_ separation performance under humid conditions than the mixed MOFs. This finding underscores the importance of precisely controlling the location of the functional groups introduced into MOFs to achieve efficient molecular separation.

**FIGURE 8:**
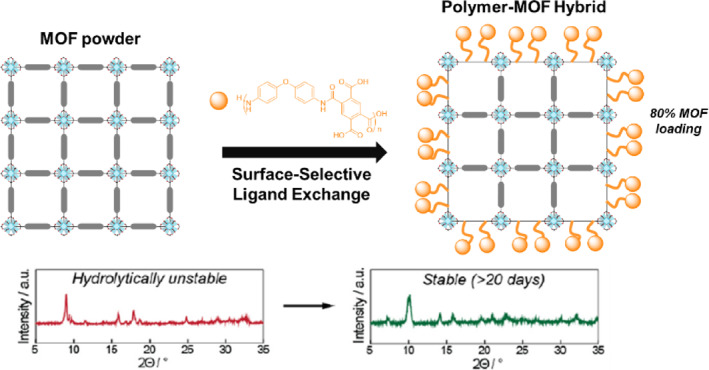
Surface‐polymer functionalization of MOFs through PSE. Adapted with permission (parts of figures) from Reference 58. Copyright 2018 American Chemical Society.

Water stability and acid–base stability are key issues in the practical industrial applications of MOFs. To enhance the stability of MOFs, strategies for blending MOFs with polymers have been extensively studied.[^9^, ^57^] In this context, the positional installation of polymeric species on the surface of MOFs was investigated by the Rzayev group in 2018 (Figure 8).^[58]^ Three representative MOFs, namely Zn‐based MOF‐5, Zn‐based ZIF‐8, and Zr‐based UiO‐66, were selected and the PSE strategy was applied to them using polymers. The synthesis of the MOF–polymer hybrids involved PSE by immersing the MOF particles in a solution of poly(amic acid) (PAA) and *N*,*N*‐dimethylformamide (DMF) at ambient temperature. In addition, the polymer PSE has also been applied to poly(dicarbomethoxyterephthalic acid‐co‐4,4‐oxydianiline) (MEPA) and poly(vinylbenzoic acid) (PVBA), with distinct functional groups in the polymer. As a result, PVBA successfully underwent ligand exchange despite its relatively weaker interactions compared to PAA. This crucial observation confirms that the incorporation of polymer chains into MOFs is driven by coordination rather than physical adsorption. Indeed, the exceptional hydrolytic stability and retention of the MOFs' porous structure (for at least 20 days) demonstrate the potential of this approach for developing MOF–polymer hybrid materials with enhanced properties.

**FIGURE 9:**
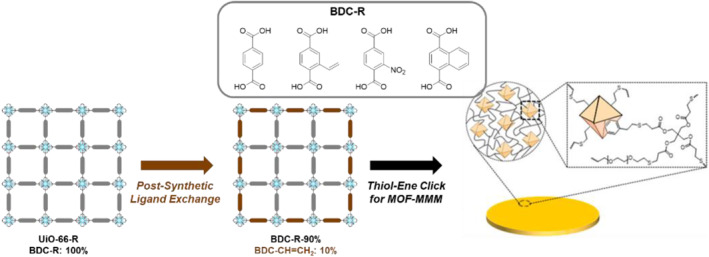
Surface vinyl group installation and thiol‐ene photo‐polymerization of MOFs and polymers. Adapted with permission (parts of figures) from Reference 11. Copyright 2020. American Chemical Society.

Positional incorporation of polymers on the surface of MOFs has been thoroughly studied by Kim et al. In 2018, Kim et al. reported thiol‐ene photo‐click chemistry for covalent connections between MOFs and polymers to construct durable MOF–polymer composites.[^9^, ^10^] The vinyl‐functionalized Zr‐based MOFs (UiO‐66‐CH=CH_2_) were used as parent MOFs to construct C–C bonds between the MOF particles and polymer chains. In a subsequent study, Kim et al. successfully incorporated a vinyl group (BDC‐CH=CH_2_) on the surface of MOF particles through the PSE approach, and thiol‐ene photo‐click reactions were performed to form covalently connected MOF–polymer composites (Figure 9).^[11]^ In addition, this study involved the synthesis of four parent MOFs, each containing distinct H, vinyl, NO_2_, and naphthyl moieties in the core. The PSE process with BDC‐CH=CH_2_ afforded four MOFs with vinyl‐functionalized shell layers. Further thiol‐ene polymerization produced four MOF surface‐polymer‐connected composites with different core functionalities (H, vinyl, NO_2_, and naphthyl). The resulting matrix membranes (MMMs) exhibited distinct gas permeability properties depending on the functional groups present in the MOF core. Notably, the MMM(30)–90%NO_2_ membrane demonstrated the highest CO_2_ permeability and CO_2_ solubility among the tested MMMs. This research shows that the selective incorporation of functional groups enables the tailoring of the properties of MOF–polymer composites for separation and related applications.

**FIGURE 10:**
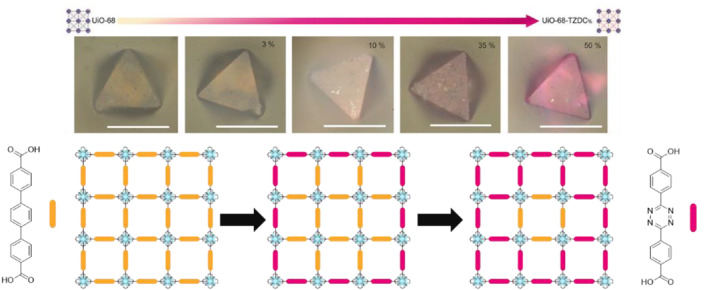
Linker distribution in Zr‐based MOFs via PSE. Adapted with permission (parts of figures) from Reference 59. Copyright 2021. American Chemical Society.

In 2021, the Gastaldo group successfully synthesized a core–shell type, Zr‐based UiO‐68‐TZDC (TZDC = tetrazine‐dicarboxylic acid) using the PSE strategy (Figure 10).^[59]^ This study aimed to investigate the impact of the distribution of the TZDC linker on photocatalytic activity. The experimental results demonstrated that a complete surface exchange exceeding 35% led to partial degradation and a decrease in photocatalytic activity. This observation suggests that excessive amounts of TZDC adversely affected the photocatalytic properties of UiO‐68‐TZDC. Conversely, a partial exchange, with approximately 10% of the surface coverage, exhibited enhanced stability in terms of photocatalytic activity. This finding indicates that a more controlled distribution of the introduced TZDC linker on the MOF surface results in improved photocatalytic performance, likely by maintaining the structural integrity and active sites of the material.

### 2.4 Further functionalization of MOFs using PSE

The PSE strategy has several key merits for MOF functionalization, including positional functionalization from the surface to the core, room‐temperature functionalization, and incorporation of metal‐chelatable ligands. This is possible because the PSE process occurs after pore formation (directional functionalization) and solvothermal synthesis (room‐temperature synthesis and chelating ligands). Numerous groups have demonstrated PSE strategies for incorporating target functionalities from these perspectives.

**FIGURE 11:**
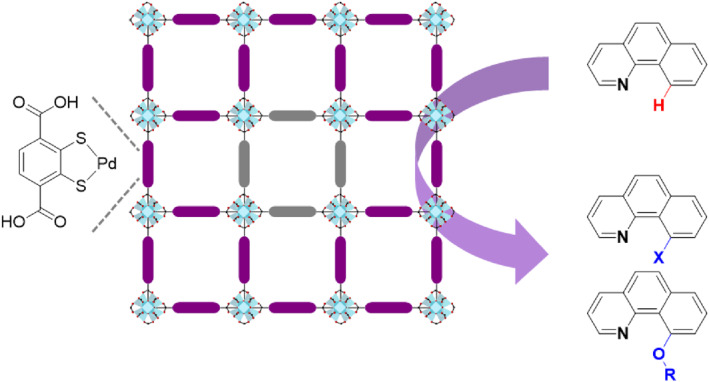
Installation of the thiocatechol group via PSE and metalation for catalysis.

In 2015, Cohen et al. employed PSE to introduce catalytic species for C–H bond functionalization (Figure 11).^[60]^ The thiocatechol group in 2,3‐dithiolato‐1,4‐benzene dicarboxylic acid (BDC‐2,3‐(SH)_2_) was installed in Zr‐based UiO‐66 MOFs using the PSE strategy, and subsequent Pd metalation was employed to prepare the UiO‐66‐PdTCAT catalyst. Thiocatechol is an efficient group for late transition metal chelation and is not suitable for direct solvothermal synthesis. Therefore, PSE provides an alternative method for preparing thiocatechol‐functionalized MOFs for palladation and C–H bond functionalization. This study represents the first example of chelation‐assisted C–H functionalization in an MOF catalyst, facilitated by a strong metal–sulfur coordination motif.

In the same year, Cohen et al. applied this thiocatechol and catechol system for the efficient synthesis of deterministic, controllable thickness, and uniform UiO‐66 films.^[61]^ Initially, nonfunctionalized parent UiO‐66 films were synthesized on fluorine‐doped tin oxide (FTO) substrates via solvothermal synthesis. Subsequently, the films were incubated at room temperature for 24 h in a pH 7 solution containing 2,3‐dihydroxyl‐1,4‐benzenedicarboxylic acid (BDC‐2,3‐(OH)_2_) to synthesize UiO‐66‐CAT films. Incorporation of the H_2_catbdc ligand was determined to be approximately 63%. Additionally, treatment with FeCl_3_ solution resulted in the formation of highly uniform films containing Fe‐catecholato species on the Zr‐MOFs. By employing the same PSE method, UiO‐66‐[FeFe] films were successfully obtained, utilizing BDC‐2,3‐(SH)_2_ as the exchange ligand. The study demonstrated the strong adhesion of the MOF films and the successful introduction of various ligands such as BDC‐2,3‐(OH)_2_ and BDC‐2,3‐(SH)_2_, paving the way for the development of functional solid‐state thin films with the desired properties.

The Matute group independently reported the incorporation of an Ir‐NHC (NHC = N‐heterocyclic carbene) metal linker into the MOF through PSE.^[62]^ A series of Ir‐NHC‐functionalized MOFs was prepared through direct solvothermal synthesis and PSE. Comparison experiments revealed that the MOF prepared from PSE exhibited a higher metal loading% and superior catalytic performance for the isomerization of allylic alcohols. The authors attributed these enhanced properties to the improved crystallinity achieved through PSE, emphasizing the utility of this strategy as an effective method for integrating metallolinkers into MOFs.

In 2018, Pan et al. reported the development of Ru and Rh photocatalyst‐incorporated Zr‐based UiO‐MOFs using a PSE strategy.^[63]^ Four distinct Ru‐ and Rh‐half‐sandwich coordination complexes were successfully integrated into MOFs and applied to the photocatalytic hydrogen evolution reaction. While molecular‐scale photocatalysts typically have limited lifespans of 5–10 h, the photocatalysts in MOFs prepared via PSE exhibit prolonged and consistent performance, maintaining high catalytic activity for up to 174 h without significant deterioration. From individual examples of the PSE process on MOFs, various transition metal catalysts for C–H bond functionalization, isomerization of allylic alcohols, and hydrogen evolution were successfully incorporated into MOFs under mild conditions while maintaining their catalytic activities.

**FIGURE 12:**
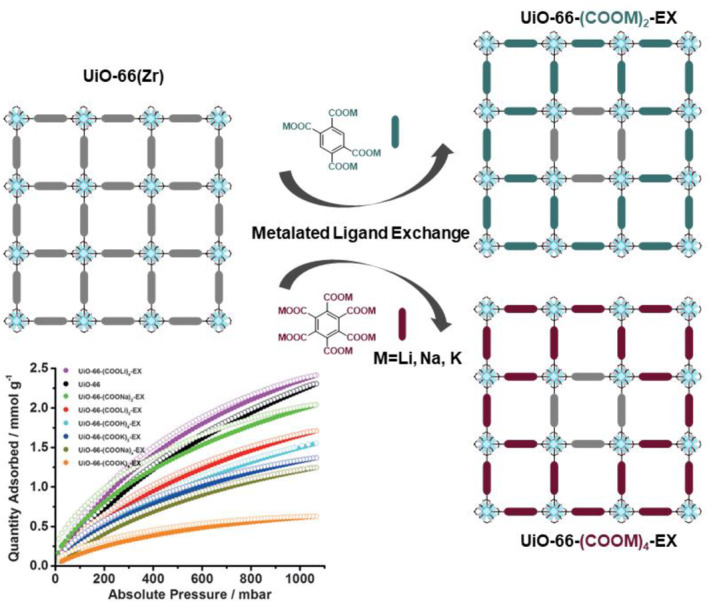
Installation of metalated ligands using PSE for CO_2_ separation. Adapted with permission (parts of figures) from Reference 64. Copyright 2015, Wiley‐VCH.

In addition to transition metals, alkali metals have been successfully incorporated into MOFs using the PSE process. In particular, chelatable carboxylate functionalization of MOFs and related alkali metalation were achieved through the PSE strategy (Figure 12).^[64]^ A series of Zr‐based UiO‐66‐(COOM)_2_ (M = Li, Na, or K) was prepared using the PSE strategy and metalation. In general, carboxylate‐functionalized MOFs are challenging to synthesize using conventional solvothermal MOF synthesis, but PSE enables the efficient synthesis of carboxylate‐functionalized MOFs. The effective exchange of over 50% of the ligands was confirmed by digested NMR, and both Li‐ and Na‐incorporated MOFs exhibited remarkable carbon dioxide separation performance. This study demonstrates the usefulness of the PSE strategy for installing chelatable ligands in coordination‐based MOF.

## 3 FUNCTIONALIZATION OF MOFs THROUGH MOF‐ON‐MOF APPROACHES

### 3.1 MOF‐on‐MOF approaches with positional functional group installations

The crystal growth of MOFs has been extended to surface applications, leading to the development of surface‐mounted MOFs (SURMOFs), particularly thin‐film MOFs.[^65^, ^66^] Notably, the surfaces of MOF crystals serve as a viable foundation for subsequent crystal growth,^[67]^ giving rise to a distinct MOF‐on‐MOF system.[^28^, ^29^, ^30^, ^31^] Notably, the MOF‐on‐MOF concept goes beyond the mere overlay of one MOF onto another; instead, it necessitates continuous coordination at the interfaces of two MOFs to establish robust interactions. Achieving interfacial lattice matching is imperative to ensure strong interactions, a feature commonly attained through the utilization of either the same MOF topology with different functionalized ligands or the same MOF topology with diverse metal ions. Furthermore, lattice mismatch can be induced through surface modification and kinetic control.^[68]^ In all scenarios, MOF‐on‐MOF systems offer a compelling avenue for the precise positional functionalization of MOFs between their core and surface components. In this section, we discuss the intricacies of functionalizing MOFs using the MOF‐on‐MOF strategy.

**FIGURE 13:**
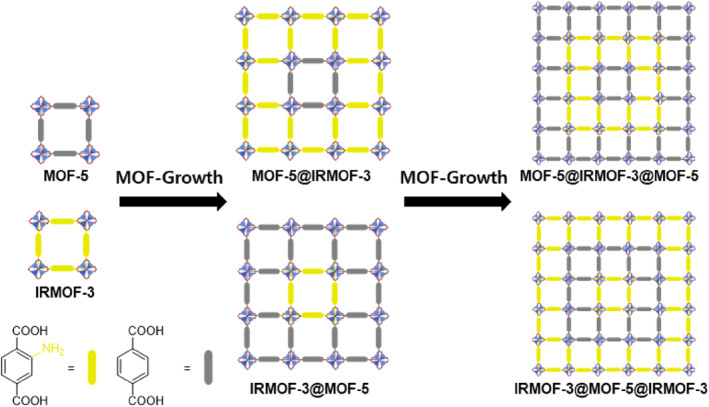
MOF‐on‐MOF strategy for Zn‐based MOFs.

In 2009, Matzger et al. reported the synthesis of Zn‐based core–shell type MOF‐on‐MOF architectures with two different ligands, BDC and BDC‐NH_2_ (Figure 13).^[69]^ The core and shell components were constructed using BDC‐based MOF‐5 and BDC‐NH_2_‐based IRMOF‐3, respectively. Furthermore, they synthesized three‐layered IRMOF‐3@MOF‐5@IRMOF‐3. Successful confirmation of this layered core–shell structure relied on optical images, taking advantage of the colorless nature of the BDC‐based MOF‐5 and the orange color of the BDC‐NH_2_‐based IRMOF‐3. This pioneering work demonstrated the direct positioning of the target functional group at specific locations, such as the core or surface.

**FIGURE 14:**
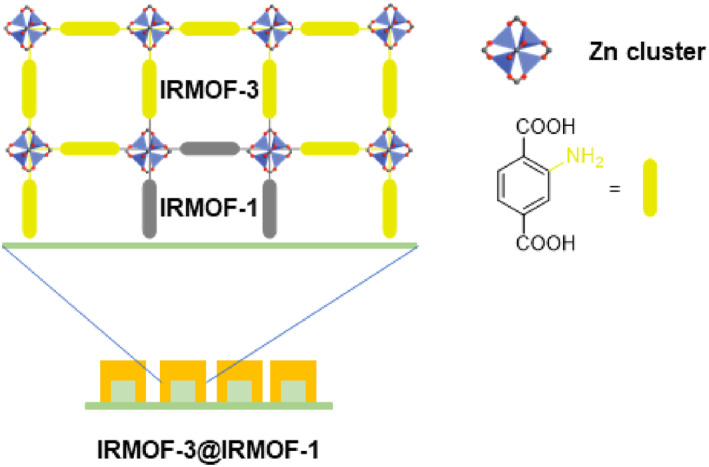
MOF‐on‐MOF strategy for thin‐film status. Adapted with permission (parts of figures) from Reference 70. Copyright 2010. American Chemical Society.

Jeong et al. also reported a similar Zn‐based MOF‐on‐MOF system with heteroepitaxial growth on a hybrid film (Figure 14).^[70]^ They again employed a combination of BDC and BDC‐NH_2_, leveraging the color differences within the Zn‐based MOF system. In Jeong's system, *N*‐ethyldiisopropylamine (EDIPA) plays a crucial role as the reagent in MOF‐on‐MOF approaches. EDIPA prevents the dissolution of core MOF crystals during the solvothermal synthesis of shell MOFs. Although PXRD pattern analysis proved inefficient for distinguishing MOF‐on‐MOF structures from physical mixtures, single‐crystal *X*‐ray diffraction (SXRD) analysis provided conclusive evidence of the epitaxy of shell MOF growth on the surface of the core MOF. This methodology further underscores the perfect positional control of the target ligands within the MOF‐on‐MOF system.

In 2011, Kitagawa et al. developed pore‐size‐controlled MOF‐on‐MOF systems for efficient molecular separation using epitaxial growth on Zn‐based MOFs.^[71]^ The primary Zn‐based paddlewheel‐type MOF system employed was Zn_2_(BDC)_2_(dabco) (dabco = 1,4‐diazabicyclo[2.2.2]octane), utilizing various combinations of functionalized dicarboxylic acid ligands, such as BDC, BDC‐NH_2_, BDC‐OH, and anthracene dicarboxylic acid (ADC). Notably, the core was constructed with the BDC ligand for large storage capability, while the shell was synthesized with ADC to impart size selectivity through narrow pores. The MOF‐on‐MOF structure was confirmed by observing the fluorescence emission from the ADC using confocal laser scanning microscopy. This pillared‐MOF‐type MOF‐on‐MOF system was successfully applied to the extraction of linear petroleum molecules from an isomer mixture, such as cetane and isocetane, even at very low concentrations (1 wt%). Despite employing the same MOF system for each core and shell, this study successfully demonstrated combinatorial approaches for MOF‐on‐MOF systems and their practical applications.

**FIGURE 15:**
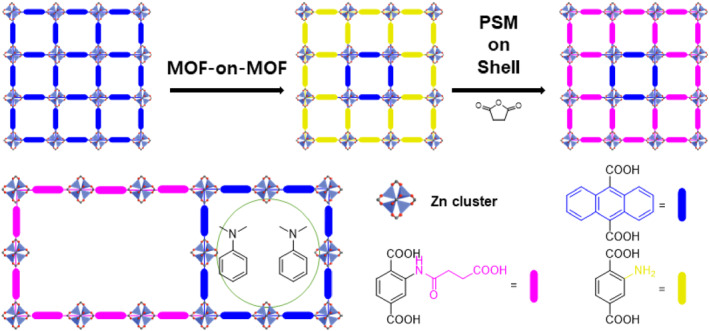
MOF‐on‐MOF combinations of Zn‐based paddlewheel‐type MOFs.

One year later, Kitagawa et al. expanded their system to MOF‐on‐MOF using a PSM approach (Figure 15).^[72]^ In this study, the core of the Zn‐based MOF was constructed with ADC, and the shell was synthesized with basic BDC‐NH_2_. However, the ADC&BDC‐NH_2_ combination in the MOF‐on‐MOF structure proved inefficient for the selective separation of benzene and *N*,*N*‐dimethylaniline (DMA). Consequently, PSM with succinic anhydride was used to introduce a carboxylic acid moiety onto the shell. Solid‐state amide bond formation enabled the acidic shell functionality in MOF‐on‐MOF. Ultimately, this ADC&BDC‐COOH combination displayed a good separation efficiency between benzene and DMA (up to an 8:2 ratio) and afforded intense exciplex fluorescence. This work successfully demonstrated targeted installation at specific positions of MOFs through a combination of MOF‐on‐MOF and PSM approaches. The functional groups of multifunctional MOFs can be controlled precisely using similar approaches.

The catalytic application of the BDC&BDC‐NH_2_ combination in the MOF‐on‐MOF strategy was investigated by Ma and Xiang in 2018.^[73]^ In the study, Zn‐, BDC‐, and triazole‐based FJU‐40 MOFs were prepared using the MOF‐on‐MOF strategy. Both BDC and BDC‐NH_2_ were utilized as core components for two distinct core–shell MOFs. Ultimately, both core–shell MOFs, namely BDC@BDC‐NH_2_ and BDC‐NH_2_@BDC, were transformed into N‐doped porous carbon (NPC) through a one‐step thermal treatment in an N_2_ atmosphere. The NPC exhibited excellent catalytic activity for the oxygen reduction reaction (ORR). Notably, BDC‐NH_2_@BDC demonstrated superior catalytic performance compared to BDC@BDC‐NH_2_, highlighting the critical role of nitrogen content control in influencing ORR properties.

**FIGURE 16:**
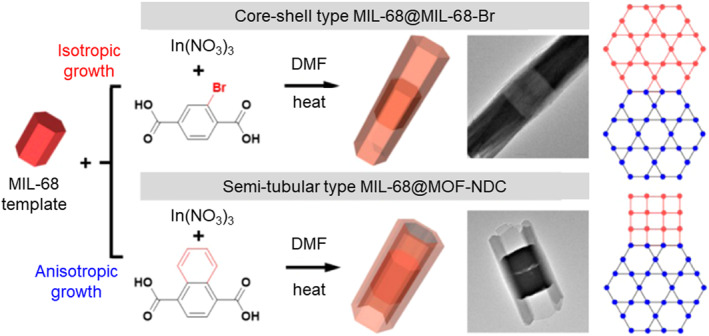
Selective crystal growth in the MOF‐on‐MOF strategy for MIL‐68(In) MOFs. Adapted with permission (parts of figures) from Reference 74. Copyright 2016. American Chemical Society.

Oh et al. conducted in‐depth investigations into positional functionalization and crystal growth in an MOF‐on‐MOF system using In‐based MIL‐68. Their meticulous study focused on the directionality of the MOF‐on‐MOF approaches. In 2016, the authors reported the isotropic and anisotropic growth of MIL‐68 crystals with BDC‐Br and naphthalene‐1,4‐dicarboxylic acid (NDC) ligands (Figure 16).^[74]^ The combination of BDC and BDC‐Br resulted in isotropic crystal growth at both ends of the crystal, forming core–shell type In‐based MIL‐68 structures. In contrast, the BDC‐NDC combination formed semi‐tubular‐connected MIL‐68 and MOF‐NDC structures. This distinction in crystal growth characteristics was attributed to the hexagonal facets observed in MIL‐68 and MIL‐68‐Br through Scanning Electron Microscopy (SEM) analysis, whereas MOF‐NDC displayed square facets. The correlation between the installation of positional functional groups and the structural features of MOFs, influencing their physical properties, was evident.

Two years later, Oh et al. expanded their approach to In‐based MIL‐68 using BDC‐NH_2_ and BDC‐NO_2_.^[75]^ Employing a similar method, they used BDC‐based MIL‐68 as the seed for the core part, with the shell part attempted using BDC‐NH_2_ or BDC‐NO_2_. Notably, the MOF‐on‐MOF‐type MIL‐68@MIL‐68‐NH_2_ and MIL‐68@MIL‐68‐NO_2_ exhibited improved crystallinity and porosity compared to pure MIL‐68‐NH_2_ and MIL‐68‐NO_2_ synthesized directly through solvothermal methods. Owing to the larger volume of the ‐NO_2_ and ‐NH_2_ functional groups, pristine MIL‐68‐NH_2_ and MIL‐68‐NO_2_ formed octahedral, spherical, and walnut‐shaped crystals, indicative of steric constraints. However, the MOF‐on‐MOF approach provided a highly crystalline MIL‐68 scaffold, resulting in excellent crystallinity and a remarkable seven‐fold increase in the Brunauer–Emmett–Teller (BET) surface area of the core–shell‐type MOFs. Crystal growth was confirmed using SEM and transmission electron microscopy (TEM), revealing an increase in the crystal length and thickness, as well as the development of a core–shell morphology. This underscores the importance of functionality not only for target applications, but also for the synthesis and fundamental properties of MOFs.

**FIGURE 17:**
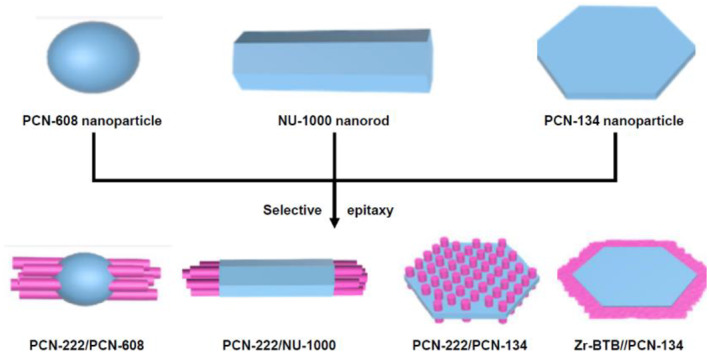
MOF‐on‐MOF functionalization of a series of Zr‐based MOFs. Adapted from Reference 76 with permission (parts of figures). Copyright 2020. American Chemical Society.

Finally, comprehensive studies on MOF‐on‐MOF systems involving Zr‐based MOFs were conducted.^[76]^ Zr‐based MOFs are known for their excellent chemical and physical stabilities, which are attributed to the high oxophilicity of zirconium and the high coordination numbers of zirconium clusters. Consequently, numerous MOFs have been reported, and their synthetic approaches and applications have been detailed.[^37^, ^54^, ^77^, ^78^, ^79^, ^80^] Zhang and his co‐workers contributed to this field by reporting a series of oriented hierarchical MOF‐on‐MOF syntheses using Zr‐based PCN MOF (PCN = porous coordination network) and NU‐1000 (NU = Northwestern University) through epitaxial growth. PCN‐222 (also known as MOF‐545), PCN‐608, and NU‐1000 are based on Zr_6_ SBU and tetracarboxylic acid ligands (Figure 17).^[76]^ Although their coordination modes are similar, the structures of the ligands and their morphologies differ significantly. In both combinations of PCN‐222 and PCN‐608 and PCN‐222 and NU‐1000, shell MOF growth at both ends of the core MOFs was observed and confirmed through SEM and TEM analyses. The porphyrin functionality in PCN‐222 was successfully installed in the core of several MOF‐on‐MOF systems, and the hydroxy group in PCN‐608 or the pyrenyl group in NU‐1000 was selectively incorporated at the surface of the MOF‐on‐MOF system. This highlights the precise control of the functionality in practical Zr‐based MOF systems achieved through MOF‐on‐MOF approaches.

**FIGURE 18:**
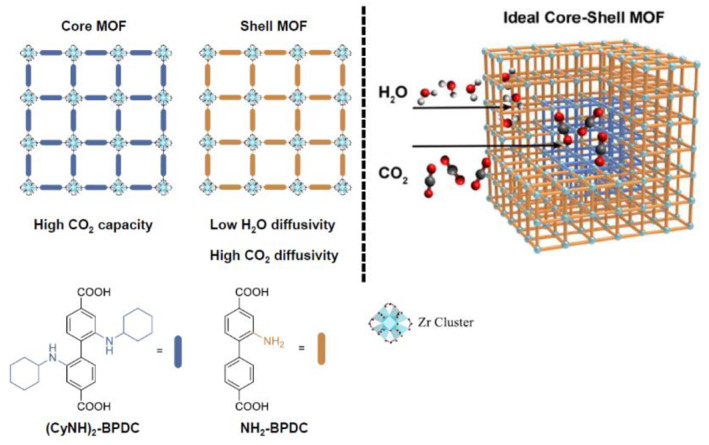
Efficient CO_2_ capture from the bifunctional MOF‐on‐MOF system. Adapted from Reference 81 with permission (parts of figures). Copyright 2023. American Chemical Society.

Rosi et al. recently pioneered an efficient CO_2_ capture system under humid conditions by utilizing a Zr‐based MOF‐on‐MOF approach (Figure 18).^[81]^ This study commenced with a systematic computational exploration to identify the core and shell MOF components that are crucial for enhanced performance. To achieve a high CO_2_ capturing ability, the incorporation of Lewis basic amine groups is deemed essential. Ensuring high stability and water resistance is imperative for efficient CO_2_ capture under humid conditions. Among the synthesized MOF‐on‐MOF‐type core–shell candidates, combinations involving BPDC‐NH_2_ and BPDC‐NH‐Cy exhibited the most promising performance and acceptable stability, in agreement with both experimental and computational findings. The core component, BPDC‐NH_2_, played a critical role in achieving high CO_2_ capacity, whereas the aliphatic cyclohexyl‐substituted secondary amine group in BPDC‐NH‐Cy contributed to low H_2_O diffusivity and high CO_2_ diffusivity concurrently. This demonstrates the significance of installing functional groups at precise positions within the MOFs. Successful collaboration between experimental synthesis and computational surveys demonstrates the potential for advancing CO_2_ capture technologies.

### 3.2 MOF‐on‐MOF approaches with positional metal ion installations

**FIGURE 19:**
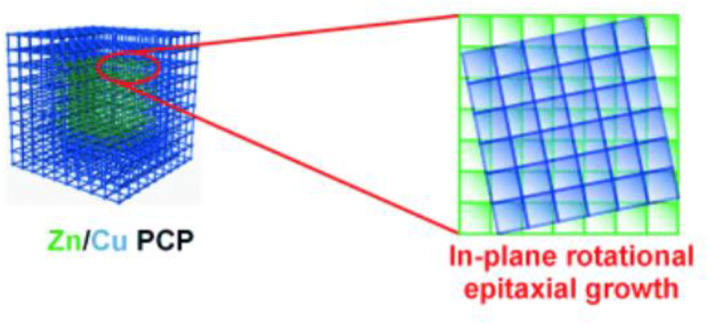
Zn‐ and Cu‐based MOFs‐on‐MOFs and their epitaxial growth. Adapted with permission (parts of figures) from Reference 82. Copyright 2009, Wiley‐VCH.

The preparation and properties of MOFs depend intricately on the characteristics of the metal ions and SBUs. Consequently, MOF‐on‐MOF strategies have been explored using various metal salts in various MOF systems. In 2009, Kitagawa et al. reported a noteworthy example involving the combination of Zn‐ and Cu‐based MOFs using the MOF‐on‐MOF strategy (Figure 19).^[82]^ The primary MOF utilized in this study was the paddlewheel‐type M_2_(BDC)_2_(DABCO) system, in which 2,6‐NDC played a significant role. Interestingly, the Cu‐based MOF could not be used as the core, preventing the attainment of the desired crystalline powder under the solvothermal conditions. However, the Zn‐based MOF successfully served as the core component, enabling subsequent shell growth with the Cu‐MOF. Face‐index analysis using SXRD confirmed that NDC and DABCO acted as growth‐directing regions for shell crystallization. Optical microscopy images successfully distinguished the colorless zinc part from the surrounding, green‐colored copper part, confirming the successful synthesis of the MOF‐on‐MOF structure.

In 2019, Rosi et al. reported the multivalent synthesis of Zr‐ and Hf‐based MOFs with various ligand combinations, and presented the concept of domain building blocks (DBBs).^[83]^ The investigation included all possible combinations of the Zr‐based UiO‐67 MOF and Hf‐based UiO‐67 MOF with ligands such as BPDC, BPDC‐I, BPDC‐NH_2_, 2, 2′‐bipyridine‐5, 5′‐dicarboxylic acid (BPYDC), and BPDC‐NH‐proline. This study extends beyond two‐layered core–shell structures by exploring three‐layered assemblies. In addition, the metalation of Pd and CuSe on MOF‐on‐MOF has been studied. This study clearly demonstrates that DBBs enable the synthesis of intricate MOF‐on‐MOF assemblies, demonstrating their potential for versatile and tailored MOF architectures. Moreover, it emphasizes the ability to introduce the desired functionality at specific positions in multivalent MOF systems.

**FIGURE 20:**
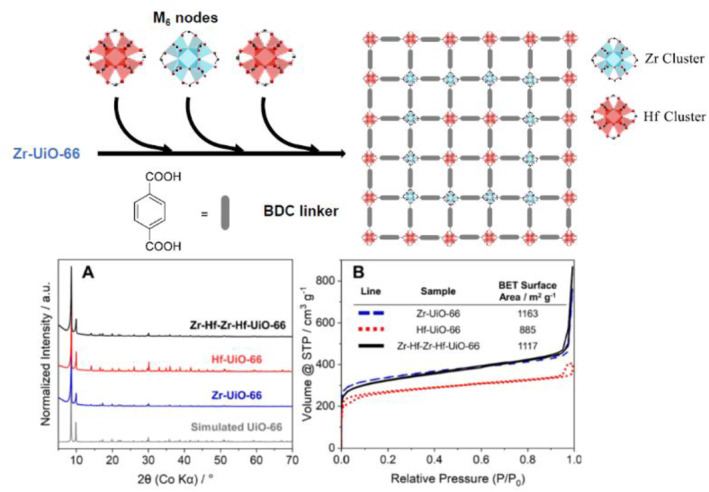
Onion‐like MOF‐on‐MOF with Zr‐ and Hf‐based MOFs: (a) PXRD and (b) N_2_ full isotherm (at 77K). Adapted with permission (parts of figures) from Reference 84. Copyright 2023, Elsevier.

Penn et al. synthesized four‐layered onion‐like UiO‐66 MOFs using Zr and Hf (Figure 20).^[84]^ Commencing with the pristine Zr‐based UiO‐66, an MOF‐on‐MOF strategy was explored using a combination of Zr, Hf, and BDC ligands. Through multiple iterations of this process, MOF‐on‐MOF structures with an onion‐like arrangement of Zr‐Hf‐Zr‐Hf‐UiO‐66 were successfully obtained. The PXRD patterns and TEM analysis revealed distinct dark and bright layers (representing Zr and Hf, respectively), confirming the successful formation of the core–shell structure. Significantly, when Hf‐UiO‐66 was employed as the core MOF, the higher oxophilicity of Hf resulted in much larger particle sizes, approximately 4–5 times larger than those of Zr‐UiO‐66 and Zr‐Hf‐Zr‐Hf‐UiO‐66. This led to a slower crystal growth, as evidenced by the higher Zr content (30%) in the four‐layered Zr‐Hf‐Zr‐Hf‐UiO‐66 MOF. This study not only achieved positional metalation but also successfully realized multilayer synthesis. Moreover, it provides crucial insights into the role of the metal composition in controlling the dynamics of crystal growth within the MOF‐on‐MOF architecture.

**FIGURE 21:**
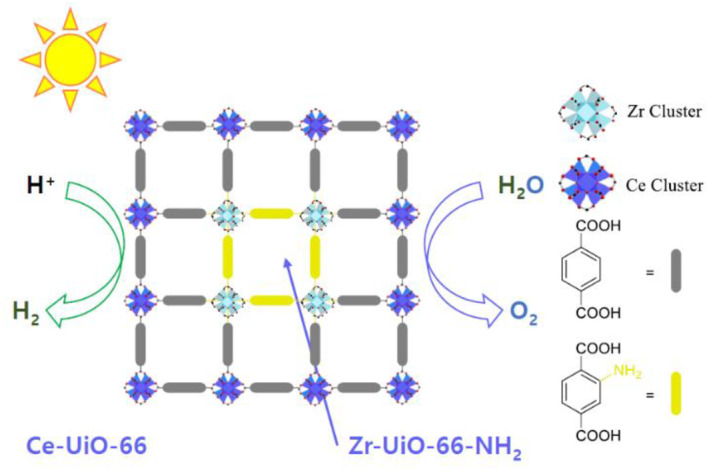
MOF‐on‐MOF strategy for the preparation of Zr‐ and Ce‐based UiO‐66 MOFs. Adapted with permission (parts of figures) from Reference 85. Copyright 2023. American Chemical Society.

In addition, different metal combinations within UiO‐66 MOFs were investigated and applied to water splitting under sunlight irradiation by the Navalon group (Figure 21).^[85]^ Both Ce‐based UiO‐66(Ce) and Zr‐based UiO‐66(Zr) were employed, and BDC and BDC‐NH_2_ were incorporated into the photocatalytic system. Among these combinations, the MOF‐on‐MOF structure formed using Zr‐based UiO‐66(Zr)‐NH_2_ as the core and Ce‐based UiO‐66(Ce) as the shell exhibits remarkable photocatalytic activity. It demonstrated 708 μmol/g for H_2_ and 320 μmol/g for O_2_ at 22 h under sunlight irradiation, owing to a lower band gap compared to individual UiO‐66 MOFs from Ce and Zr. The MOF‐on‐MOF structure was confirmed through EDX, TEM, and FT‐IR spectroscopy. Notably, the combination of the Zr core/Ce shell showed superior performance compared to the Ce‐core/Zr shell structure. This result suggests that UiO‐66(Ce) was not completely stable in the presence of the acidic ZrCl_4_ reagent under the second solvothermal condition. Therefore, this study clearly demonstrates the efficient catalytic application of MOF‐on‐MOF structures, emphasizing the importance of judicious choices in MOF‐on‐MOF synthesis based on the properties and characteristics of each MOF component.

### 3.3 Lattice‐mismatched MOF‐on‐MOF approaches with different metals

**FIGURE 22:**
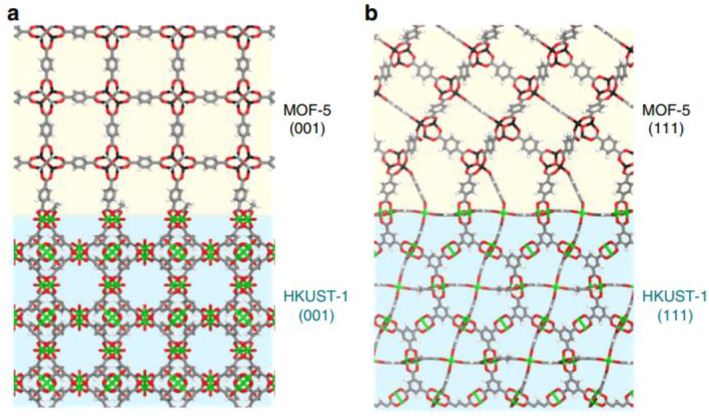
MOF‐on‐MOF composites of Zn‐based MOF‐5 and Cu‐based HKUST‐1: (a) HKUST‐1 (001)/MOF‐5 (001) and (b) HKUST‐1 (111)/MOF‐5 (111). Adapted from Reference 86 with permission (parts of the figures). Copyright 2019 Nature Publishing Group.

Interfacial connections between MOFs with the same topology but different functional groups or metal ions are well established. However, the interfacial connections between MOFs with different topologies or lattice‐mismatched MOF‐on‐MOF structures are feasible for specific combinations. A collaborative research team led by Moon and Kim reported the successful synthesis of the predicted MOF‐on‐MOF structures using two different MOF systems (Figure 22).^[86]^ They experimentally verified that Cu‐based HKUST‐1 and Zn‐based MOF‐5 possessed precisely matched square (001) surfaces, as initially predicted computationally. Furthermore, MOF‐5 and UiO‐66 did not undergo crystal growth in the MOF‐on‐MOF configuration because of the absence of matching surfaces. This research not only enabled the computational prediction of whether the desired MOF could be successfully synthesized in the form of MOF‐on‐MOF but also identified an algorithm for synthesizing optimized MOF‐on‐MOF structures.

In 2014, Kang et al. attempted an experimental MOF‐on‐MOF approach using two different MOFs. They combined Cr‐based MIL‐101 and Zr‐based UiO‐66 using the same BDC ligand used in the MOF‐on‐MOF concept.^[87]^ The obtained MOFs were analyzed using PXRD, SEM, and TEM. Overall, the core–shell MOF exhibited two mesopores and demonstrated a higher BET surface area than the single MOFs, indicating improved adsorption properties. This material has also been used for hydrogen storage. However, the study did not fully conduct detailed interfacial and positional analyses.

In 2017, Kitagawa and co‐workers established an MOF‐on‐MOF system involving Zr‐based UiO‐66‐NH_2_, Ti‐based MIL‐125‐NH_2_, Sc‐based MOF‐76, and Cr‐based MIL‐101, utilizing a lattice‐mismatching internal extended growth method rather than an epitaxial growth method.^[88]^ Notably, they employed a polyvinylpyrrolidone (PVP) polymer coating after preparing the first monodisperse MOF. Although the specific role of the PVP layer warrants careful investigation, this methodology presents a general protocol for preparing various MOF‐on‐MOF systems under lattice‐mismatching conditions. This study proposes a modular 3D core–satellite MOF architecture that deviates from conventional core–shell MOF composite materials.

**FIGURE 23:**
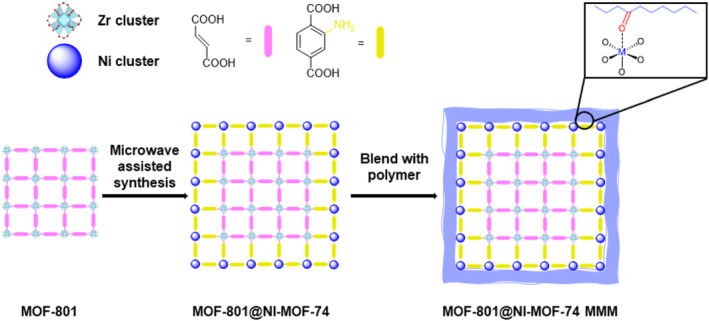
MOF‐on‐MOF between MOF‐801 and MOF‐74. Adapted with permission (parts of figures) from Reference 89. Copyright 2020. American Chemical Society.

In 2020, Li et al. combined Zr‐based MOF‐801 from aliphatic fumaric acid and Ni‐based MOF‐74 from aromatic BDC–(OH)_2_ ligands for further blending with polymers for gas separation applications (Figure 23).^[89]^ Following the synthesis of core MOF‐801 according to the literature, the shell MOF‐74 was synthesized. MOF‐74 features irregular intermediate pores, providing a textured surface and increased interfacial area. The hydroxy groups on the shell, along with the open metal sites, exhibited strong interactions with the polymer matrix, facilitating successful coating of the shell layer. This unique MOF demonstrated robust interactions that were unattainable with conventional MOF‐on‐MOF growth owing to the presence of open metal sites. Positional installation of functional groups on the shell has emerged as crucial for targeted applications and the formation of MOF‐on‐MOF architectures.

### 3.4 Lattice‐mismatched MOF‐on‐MOF approaches with different ligand lengths and coordination

In isoreticular MOF‐on‐MOF systems, lattice‐mismatched structures can be achieved with the same metal sources and coordination modes but different ligand lengths. For instance, in 2014, the Wöll group utilized a Cu‐BDC MOF system on an Au substrate by employing the SURMOF and MOF‐on‐MOF concepts.^[90]^ The dicarboxylic acid ligands, BDC (with a single benzene ring), NDC (with a naphthalene ring), and BPDC (with a biphenyl group), were combined at varying dicarboxylic acid lengths to obtain lattice‐mismatched MOF‐on‐MOF structures. A combination of NDC‐BPDC and BDC‐NDC‐BPDC was adopted for the MOF‐on‐MOF thin‐film system, resulting in hierarchical porous MOFs with distinct pore sizes in each layer, enabling the selective storage of size‐selective nanoparticles. Despite the significant lattice mismatch, this pioneering work successfully demonstrated a useful methodology for target installation at specific positions with different ligand lengths.

**FIGURE 24:**
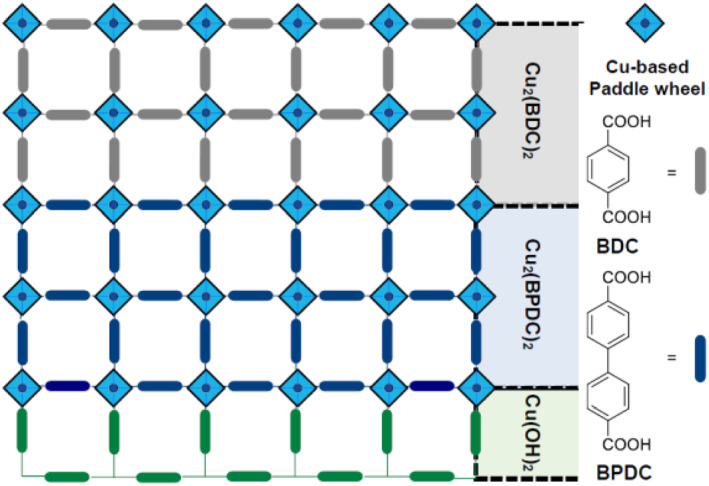
MOF‐on‐MOF for multilayered thin‐film formation.

Expanding on the lattice‐mismatch concept in 2019, Takahashi et al. applied the Cu‐BDC‐type MOF‐on‐MOF system to a Cu(OH)_2_ template within a thin film, which was utilized for silver nanoparticle preparation (Figure 24).^[91]^ This work presents the application of MOFs in multifunctional porous coating systems, relying on the computational analysis of lattice matching between Cu(OH)_2_ and Cu‐BDC MOFs. By substituting the BDC ligand with BPYDC (with a bipyridyl moiety) during synthesis, additional silver cations could be placed on the shell of the MOF‐on‐MOF thin film through coordination between the silver cations and bipyridyl sites. The subsequent reduction process resulted in the formation of AgNPs in a specific shell of the MOF‐on‐MOF. Cu‐BPYDC could not be obtained on the Cu(OH)_2_ layer through direct synthesis, therefore, a Cu‐BPDC layer (core) was necessary for successful synthesis. This demonstrated the introduction of two distinct functions at specific positions in the MOF‐on‐MOF strategy, where Cu‐BPDC in the bottom part provided a stable bedding layer, and Cu‐BPYDC in the upper part enabled a catalytic site with a specific Ag loading.

Two years later, Takahashi et al. developed layered Cu MOF systems on thin films using an MOF‐on‐MOF strategy for electronic and photonic applications.^[92]^ In this system, four‐layered structures were prepared: Cu(OH)_2_/Cu‐BPDC/Cu‐NDC/Cu‐BDC and Cu(OH)_2_/Cu‐BDC/Cu‐NDC/Cu‐BPDC. The relationship between the lattice mismatch ratios and the orientations of MOF‐on‐MOF with epitaxial interfaces was carefully studied, providing a general method for preparing multifunctional MOF‐on‐MOF thin films.

**FIGURE 25:**
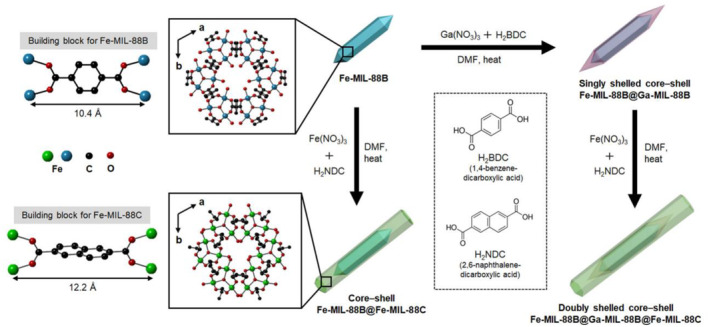
MOF‐on‐MOF strategy for synthesizing MIL‐88B from BDC and MIL‐88C from NDC. Adapted with permission (parts of figures) from Reference 93. Copyright 2020. American Chemical Society.

In a study published in 2020, Oh et al. investigated lattice‐mismatched MOF‐on‐MOF structures with Cu‐based MOFs, Fe‐based MOFs, and Ga‐based MOFs (Figure 25).^[93]^ The anisotropic crystal growth of MIL‐88(Fe) was investigated through detailed mechanistic observations. In the MIL‐88(Fe) system, the BDC ligand of length 10.4 Å forms the MIL‐88(Fe)‐B structure, while the NDC ligand of length 12.2 Å produces the MIL‐88(B)‐C structure. Systematic investigations were conducted on MIL‐88(Fe)‐B/MIL‐88(Fe)‐C and MIL‐88(Fe)‐B/MIL‐88(Ga)‐B, and the study was successfully expanded to include double‐shelled structures such as MIL‐88(Fe)‐B/MIL‐88(Ga)‐B/MIL‐88(Fe)‐C.

In another study of lattice‐mismatched MOF‐on‐MOF systems, Xu et al. studied Zr‐based UiO MOFs.^[94]^ UiO‐66 (from BDC), UiO‐67 (from BPDC), and UiO‐68 (from the terphenyl system) were combined with Au and Pd nanoparticles to form spherical Sandwich structures. In this study, shell layers were utilized to protect the Au and Pd nanoparticles and to impart selectivity. Metal nanoparticles can be installed in a specific layer using this MOF‐on‐MOF strategy. The resulting MOF‐on‐MOF/metal nanoparticle system was used to reverse the water‐gas shift reactions.

Chaemchuen and Verpoort reported on the MOF‐on‐MOF concept using a Zr‐based UiO‐66 MOF (from BDC) and UiO‐67‐BPY (from BPYDC).^[95]^ The core was composed of a stable UiO‐66 MOF, and the shell was decorated with Lewis basic UiO‐67‐BPY for catalytic applications. The synthesized MOF‐on‐MOF architecture exhibited PXRD patterns resembling UiO‐66 and UiO‐67, showing BET surface values between UiO‐66 and UiO‐67‐BPY. The well‐dispersed BPY sites on the shell were successfully applied to catalytic Knoevenagel condensation between aldehydes and malononitriles. The specific loading of catalytic sites on the shell layer prevented mass diffusion limitations, and the strong‐core MOF enabled a recyclable heterogeneous system.

**FIGURE 26:**
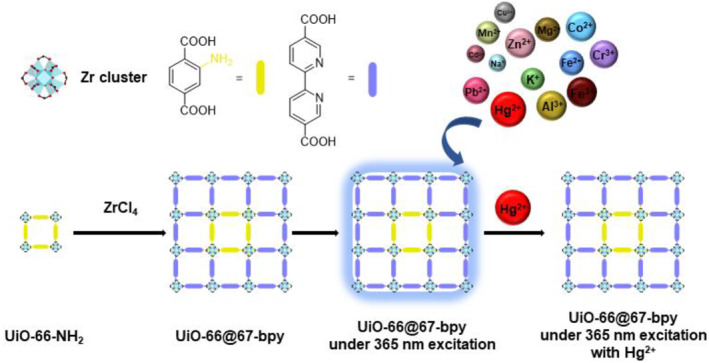
Raspberry‐shaped MOF‐on‐MOF prepared from UiO‐66 and UiO‐67‐BPY. Adapted from Reference 96 with permission (parts of the figures). Copyright 2022. American Chemical Society.

The morphology of the UiO‐MOF‐type MOF‐on‐MOF was investigated by Liu et al. (Figure 26).^[96]^ The second crystal growth of UiO‐67‐BPY was performed on the core UiO‐66‐NH_2_. The researchers obtained raspberry‐shaped MOF‐on‐MOF materials, as evidenced by SEM, from this system by optimizing the reaction time, mode, and concentration. MOF‐on‐MOF displayed stable fluorescence and desirable sensing selectivity for mercury ions. A computational study revealed charge transfer between the mercury ion and the MOF‐on‐MOF ligand, highlighting a synergistic effect.

Liang et al. expanded the application of the combination of UiO‐66 and UiO‐67‐BPY in the MOF‐on‐MOF strategy to rhodium catalysis and enzyme‐directed modification.^[97]^ UiO‐67‐BPY was initially prepared and used as the core, followed by metalation with Rh at the bipyridyl sites of the MOFs. The UiO‐66 shell was added via a second solvothermal‐synthesis step. The core‐placed Rh‐BPY was utilized for NAD(P)H regeneration and the UiO‐66 shell was used to specifically immobilize His‐tagged lactate dehydrogenase (LDH), which served as a protective layer for the LDH and Rh‐BPY species to prevent mutual inactivation. The specific positions of Rh‐BPY and LDH were critical for catalysis in this MOF‐on‐MOF system. Target functionalization was successfully achieved using this smart multifunctional material.

Building on the contributions of Zhang et al. to Zr‐based MOFs for lattice‐matched MOF‐on‐MOF systems in 2020, lattice‐mismatched combinations were examined (Figure 17).^[76]^ PCN‐134 is a Zr‐based MOF derived from a ligand mixture of a tetracarboxylic acid ligand and tricarboxylic acid with Zr_6_ nodes. A MOF‐on‐MOF strategy of PCN‐134 with tetracarboxylic acid‐based PCN‐222 and tricarboxylic acid‐based Zr‐BTB MOF was developed. For PCN‐222/PCN‐134, PCN‐222 crystals developed on the basal plane of PCN‐134. Conversely, when BTB was employed for MOF growth, Zr‐BTB crystals grew at both ends of the structure. These discrepancies can be attributed to the lattice mismatch between the core and the growing shell MOF, which results in epitaxial growth occurring exclusively on specific surfaces. This study clearly demonstrated a variety of combinations of Zr‐based MOFs through lattice‐matched and lattice‐mismatched cases.

## 4 CONCLUSION AND OUTLOOK

Functionalization plays a pivotal role in enhancing the versatility and applicability of MOFs, distinguishing them as unique porous materials. The ability to introduce specific functionalities at precise positions within the MOF structure imparts tailor‐made properties, enabling myriad applications and the preparation of multifunctional and smart materials. MOFs offer exceptional platforms for gas storage, separation, catalysis, and sensing, and the strategic placement of functional groups enhances their performance in these domains. This inherent flexibility in MOF design, which enables the incorporation of diverse ligands and metal nodes, positions them as promising candidates for addressing a wide range of challenges in material chemistry.

In the realm of MOF functionalization, PSE strategies have emerged as powerful tools for introducing specific functionalities. PSE enables the replacement of existing ligands with the desired functional groups, thereby fine‐tuning MOF properties. This approach provides a means to overcome the limitations associated with the initial synthesis (i.e., solvothermal conditions) and offers a flexible route for adapting MOFs to diverse applications. Moreover, the newly added functionalities are generally placed on the surface (i.e., the outer side) of MOFs through this invasive method.

MOF‐on‐MOF strategies also offer a unique avenue for achieving precise, spatially controlled functionalization on the surface of MOFs through additory methods. By growing one MOF layer upon another, this approach facilitates the creation of multifunctional architectures with distinct layers tailored for specific purposes. Various position‐specific functionalizations have been achieved using MOF‐on‐MOF strategies, including catalysis, sensing, and molecular separation.

In the context of functionalized MOFs and target applications, PSE emerges as a potent method for imbuing MOFs with reactive or coordinative functional group decorations. Given that MOF frameworks are composed of metal‐ligand coordination, the introduction of coordinative groups during solvothermal synthesis can yield competitive effects. PSE offers a viable alternative for incorporating these groups into MOFs. Consequently, the exploration of MOFs featuring coordinative functional groups has garnered significant attention, particularly in catalysis and polymerizations. Beyond reactive group installations, surface decoration with external ligands through PSE unlocks intriguing applications in MOFs. Gas separation properties are finely tuned by core and shell positions, while polymerizations are confined to the MOF surface. Notably, the relatively facile control of functionalization ratios underscores a pivotal aspect of PSE in MOFs. Leveraging the PSE strategy, advancements in color control and molecular sensing have been achieved in MOFs.

Meanwhile, the fabrication of MOF‐on‐MOF architectures via various solvothermal synthetic routes presents an avenue for creating composite structures. Although MOF‐on‐MOF constructions may not yield environments as diverse as those obtained using metal‐chelatable ligands, they preserve the structure and properties of individual MOFs. This preservation allows for the prediction of both chemical and physical properties. Consequently, the exploration of gas adsorption applications has been a focal point of research utilizing the MOF‐on‐MOF strategy, including investigations into thin‐layer formations. Furthermore, MOF‐on‐MOF approaches have facilitated the introduction of dual catalytic activities and enhanced sensing properties into MOFs, underscoring the versatility and potential of this synthetic methodology.

Despite significant advancements in specific positional functionalization through PSE and MOF‐on‐MOF strategies, certain limitations and challenges persist. In the PSE approach, the success of ligand‐exchange reactions may be influenced by the stability of the MOF, and achieving quantitative ligand exchange through analytical methods can be challenging. Moreover, the potential loss of crystallinity or structural integrity during the exchange process may limit the applicability of this strategy to certain MOF systems. Additionally, PSE is closely associated with structural defects in MOFs. Distinguishing between ligand insertion at defect sites and ligand exchange with existing ligands remains challenging, warranting further detailed analysis.

Regarding MOF‐on‐MOFs, achieving precise control over the interface between core and shell MOFs poses a critical challenge. Lattice‐matching conditions and crystallographic orientations are crucial factors influencing the success of the MOF‐on‐MOF strategy. Moreover, exploring the scalability of this approach and its application in large‐scale syntheses is essential. Additionally, the limitation of crystallization size also restricts MOF‐on‐MOF approaches in developing multi‐layered MOFs. Therefore, synthesizing complex multilayered MOF architectures using MOF‐on‐MOF may also present challenges in terms of reproducibility and scalability.

In the PSE and MOF‐on‐MOF cases, the recombination of specific functionalizations should be carefully considered and examined. As the coordination bonds between the SBU and ligands are reversible, solid diffusion is still possible. The target ligands on the surface should be oriented randomly to random distributions on coordinative conditions (i.e., solvent‐assisted dissolution of the ligand). Therefore, the final thermodynamic states of these layered materials must be carefully investigated.

In addition to the ligand‐matched PSE and MOF‐on‐MOF strategies, positional functionalizations through core‐shell composite type materials have also been extensively studied. For example, significant progress has been made in biomolecular functionalizations on MOFs. Various biomolecules such as DNA, carbohydrates, and proteins have been successfully incorporated into MOFs, particularly at the surface due to their larger size compared to typical MOF pores. The functionality of each biomolecule at the surface of MOF particles is interconnected with the porosity of MOFs.[^98^, ^99^, ^100^, ^101^, ^102^]

The future of specific positional functionalization in MOFs will involve addressing these challenges and exploring new frontiers. Advances in computational techniques and machine learning could play a pivotal role in predicting the optimal ligand designs for PSE and guiding the growth of MOFs in MOF‐on‐MOF systems. The integration of these strategies with other emerging techniques such as defect engineering and PSM may offer synergistic approaches for achieving enhanced control over MOF properties and multifunctional smart materials. Additionally, the positioning of MOF particles within the bulk scale is currently a crucial area of recent research. Several techniques, including flow chemistry and magnetism, have been developed to precisely control the position and alignment of MOF particles within specific plates or bulk materials.[^103^, ^104^, ^105^] Ultimately, achieving precise control over functional groups within MOFs, the positional control of functionalities in MOFs, and the positioning of MOF particles on bulk scales are essential for controlling the functions of MOFs.

## CONFLICT OF INTEREST STATEMENT

The authors declare no conflicts of interest.

## Data Availability

The data that support the findings of this study are available from the corresponding author upon reasonable request.
